# Comparison of four bone substitute types in sinus augmentation with perforated Schneiderian membrane: An experimental study

**DOI:** 10.1002/JPER.24-0663

**Published:** 2025-04-02

**Authors:** Jung‐Tae Lee, Seok Hyun Lee, Baek Sun Choi, Sungtae Kim

**Affiliations:** ^1^ Department of Periodontics One‐Stop Specialty Center Seoul National University Dental Hospital Jongno‐gu Seoul Republic of Korea; ^2^ Xenograft Development Team Tissue Regeneration Institute Osstem Implant Co. Ltd. Gangseo‐gu Seoul Republic of Korea; ^3^ Department of Periodontology Dental Research Institute Seoul National University School of Dentistry Jongno‐gu Seoul Republic of Korea

**Keywords:** bone graft, bone regeneration, fibrin tissue adhesive, maxillary sinus, ossification

## Abstract

**Background:**

Collagenated bovine bone mineral (CBBM) is used for maxillary sinus elevation‐based augmentation. We compared collagen degradation in cross‐linked (CL) CBBM and non‐cross‐linked (NCL) CBBM (in vitro) and preclinical results in CBBM‐CL, CBBM‐NCL, deproteinized bovine bone mineral (DBBM), and DBBM with fibrin sealant grafts (in vivo) after repair of perforated Schneiderian membrane (SM).

**Methods:**

Collagenase‐based degradation rates of CBBM‐CL and CBBM‐NCL were compared in vitro and in vivo (CBBM‐CL, CBBM‐NCL, DBBM, and DBBM with fibrin glue; *n* = 12 defects per group). After bilateral maxillary SM perforation (5 mm) during sinus elevation, we performed bone substitute‐based augmentation. At 4 and 12 weeks postoperatively, volumetric, histologic, and histomorphometric analyses were performed.

**Results:**

Complete degradation and 33% retention of collagen content after 24 and 72 h were noted in CBBM‐NCL and CBBM‐CL, respectively. CBBM‐NCL demonstrated significant differences in total augmentation volume (TAV) compared to DBBM and DBBM with fibrin glue, as well as in new bone volume (NBV) compared to DBBM with fibrin glue. At 12 weeks, significant differences were observed between CBBM‐NCL and DBBM in NBV. There were no significant differences across all groups in vertical bone increase in 4 and 12 weeks. DBBM and DBBM with fibrin glue showed more irregularly shaped patterns than CBBM‐CL and CBBM‐NCL. At 12 weeks, ossification progressed in all groups. At 4 weeks, DBBM with fibrin glue seemed to demonstrate early ossification at the perforation site in histological observations compared to DBBM alone; however, no differences were observed at 12 weeks.

**Conclusion:**

CBBM for perforated SM repair confers bone stability and ossification. CBBM‐CL was noninferior to CBBM‐NCL in volume stability. The findings indicate that the CL collagen in CBBM‐CL contributes to enhanced graft stability over time. Fibrin glue appeared to have a positive effect on early ossification in histological evaluations, but this effect was not evident at 12 weeks.

**Plain Language Summary:**

Collagenated bovine bone mineral (CBBM) is used for maxillary sinus elevation‐based augmentation. We compared collagen degradation in cross‐linked (CL) CBBM and non‐cross‐linked (NCL) CBBM (in vitro) and preclinical results in CBBM‐CL, CBBM‐NCL, deproteinized bovine bone mineral (DBBM), and DBBM with fibrin sealant (in vivo) after repair of perforated Schneiderian membrane (SM). After bilateral maxillary SM perforation (5 mm) during sinus elevation, we performed bone substitute‐based augmentation. At 4 and 12 weeks postoperatively, volumetric, histologic, as well as histomorphometric analyses were performed. Based on this study, CL collagen in CBBM‐CL contributes to enhanced graft stability over time. Fibrin glue appeared to have a positive effect on early ossification in histological evaluations, but this effect was not evident at 12 weeks.

## 1 INTRODUCTION

Maxillary sinus pneumatization confers unfavorable conditions for implant placement; however, successful implants necessitate sufficient bone height and width.^1^ A previous study reported that the mean residual bone height in the posterior maxilla was approximately 5 mm prior to sinus augmentation procedures.^2^ Sinus elevation procedures include the crestal and lateral approaches. At the consensus conference on sinus elevation, residual bone height was classified as A–D, and the lateral approach was recommended in cases with insufficient alveolar bone (1–6 mm).^3^ Sinus elevation‐associated complications include systemic disease, medications, anatomy, surgical procedures, sinus pathology, and infection, and so forth.^4^ Zijderveld et al. reported that Schneiderian membrane (SM) perforation was the most frequent complication (11%) during sinus elevation,^5^ whereas another study ascertained that the incidence of SM perforation was 23.5%.^6^ SM perforation is attributable to insufficient peri‐SM dissection and vigorous instrumentation,^7^ and risk factors are categorized into (1) anatomical risks (sinus septa and SM thickness), (2) surgical risk (technique and piezoelectric instrumentation), and (3) pathological risk (sinusitis).^6^ Hernandez‐Alfaro et al. showed that the size of the SM perforation affected implant survival.^8^ Nolan et al. reported a higher risk of bone‐grafting failure (three times) and sinusitis (six times) with SM perforation. Thus, an SM perforation must be managed immediately. Perforations of 5–10 mm can be repaired using a collagen membrane, fibrin glue, biologic agents, and so forth, whereas perforations larger than 10 mm warrant delaying the bone grafting.^9^

Sinusitis can be caused by bone particles that escape into the maxillary sinus through the perforated membrane and block the ostium.^10^ Introduced in 2008, collagenated bovine bone mineral (CBBM), which comprises deproteinized bovine bone mineral (DBBM) with 10% collagen,^11^ serves as an effective bone substitute and has been extensively utilized in clinical procedures such as alveolar ridge preservation (ARP).^12^, ^13^ Han et al. applied CBBM to buccal dehiscence defects^14^ and, following sinus elevation with an intentionally perforated SM, CBBM favorably augmented volume stability.^15^

Collagen, comprising a bone particle matrix in CBBM, plays an important role in maintaining the shape of the grafted area. Over time, the graft volume may decrease rapidly owing to collagen degradation.^16^ Bone particle–collagen cross‐linking in CBBM was developed to mitigate this limitation. An et al. showed that, compared to a non‐cross‐linked (NCL) bone substitute, cross‐linked (CL) collagen‐integrated xenogeneic bone blocks showed favorable regenerative healing in rabbit calvaria and also noted that CBBM‐CL possesses the following advantages over CBBM‐NCL: (1) enhanced volumetric stability, (2) improved mechanical properties, (3) support for osteogenesis, and (4) reduced risk of collapse.^17^ However, compared to studies of collagen membranes, there is limited research on CL‐CBBM.^18^, ^19^

Introduced for bone adhesion and hemostasis of wounded tissues, fibrin glue functions via a tissue damage‐induced mechanism, where fibrinogen exuding from capillaries interacts with blood coagulation components to form fibrin. The clinical advantages of fibrin glue include no need for heat and pressure application, platelet‐based coagulation, biocompatibility, and suitable resorption.^20^ Compared to controls, a test group that used fibrin glue had a considerably lower inflammatory index.^21^ Romanos and Strub demonstrated the biocompatibility of fibrin glue that mitigates inflammatory reactions during healing.^22^

In this study, we evaluated the collagen degradation of CBBM‐CL and CBBM‐NCL (in vitro) and compared the augmented volume outcomes of DBBM, DBBM with fibrin glue, and two types of CBBM (CL and NCL) in the repair of perforated SM in rabbits (in vivo). We hypothesized that, for sinus augmentation in perforated SM, CBBM‐CL would demonstrate volume stability comparable to CBBM‐NCL, DBBM, and DBBM with fibrin glue.

## 2 MATERIALS AND METHODS

### 2.1 In vitro collagen degradation of collagenated bone

We procured CBBM‐CL* and CBBM‐NCL†. To evaluate the rate of collagen degradation in collagenated bones, a digestion solution was prepared by dissolving *Clostridium*‐derived collagenase in phosphate‐buffered saline (pH 7.4). After recording the initial mass of each sample, 100 mg per sample was immersed in 1 mL of 2 mg/mL collagenase solution for 1, 3, 6, 24, and 72 h. Samples were collected at each predetermined time point, washed with distilled water and centrifuged at 1000 rpm for 5 min to remove the remaining supernatant, and then dehydrated overnight by lyophilization. The dry weights of the samples were recorded, and the percentage of the remaining mass at each timepoint was calculated.

### 2.2 Experimental animals

Twenty‐four healthy male New Zealand white rabbits (weight 2.5–3.0 kg) were obtained, acclimatized for 2 weeks before the experiments, housed individually under standard conditions, and provided a regular diet and unlimited access to water. The study protocol was reviewed and approved (OST‐IACUC2306) by the Institutional Animal Care and Use Committee (IACUC) of the testing facility.

### 2.3 Study design

Based on previous studies,^15^, ^23^ bilateral SM perforation (5 mm) was performed using a scalpel blade in the maxilla of each rabbit after sinus elevation, and the rabbit was assigned to one of the groups (*n* = 12 defects per group) for receiving the same volume of bone graft: (a) CBBM‐CL, (b) CBBM‐NCL, (c) DBBM‡, and (d) DBBM with fibrin glue§, respectively.

### 2.4 Surgical procedure

**FIGURE 1:**
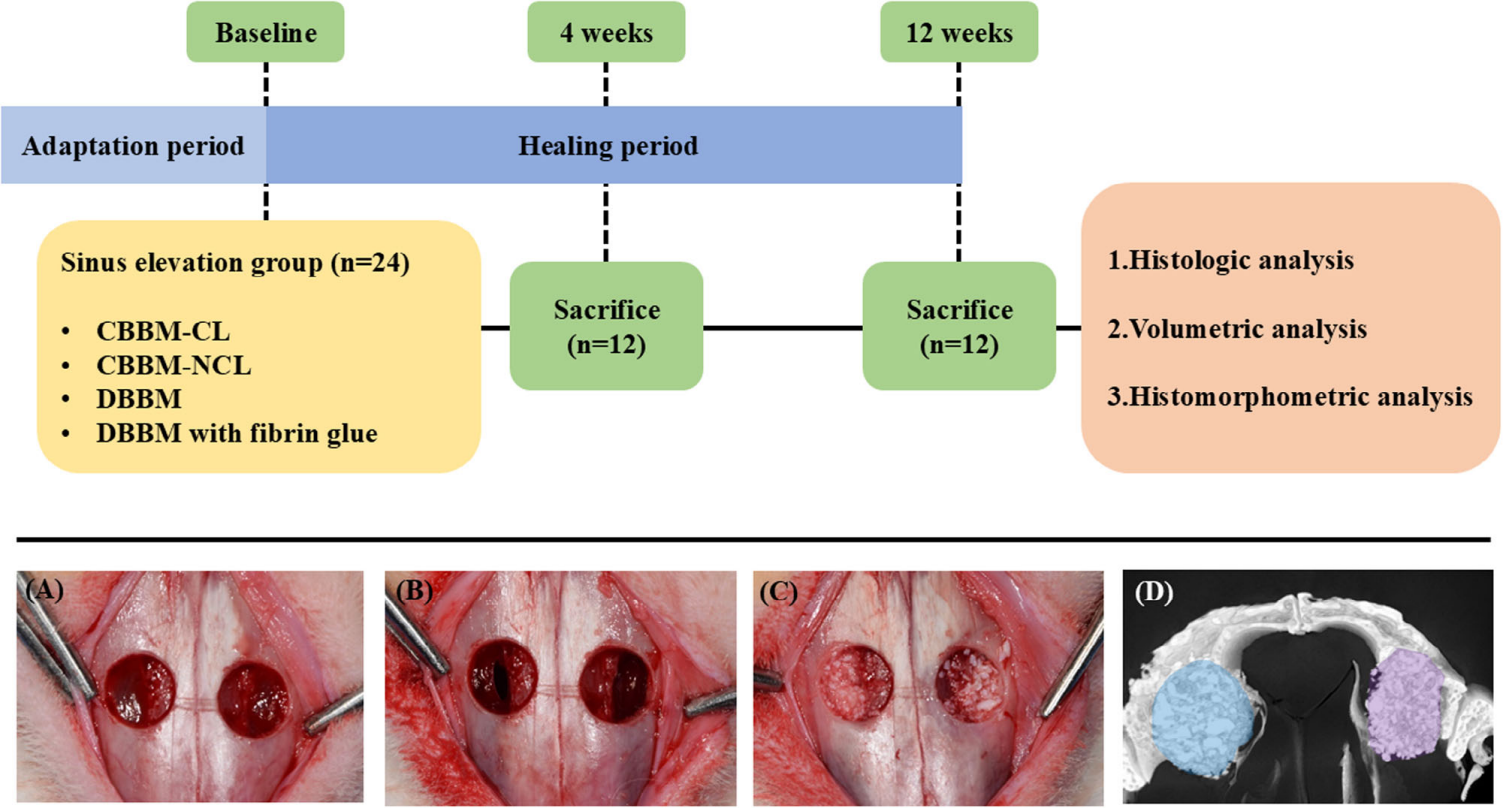
Flowchart and clinical photographs of surgical procedures. (A) Circular bony windows (diameter 7.0 mm) were created. (B) Intentional perforation (length 5.0 mm) in Schneiderian membrane on each side. (C) Grafting of 60 mg CBBM. (D) Color‐coded 3D‐reconstructed microcomputed tomographic images. CBBM, collagenated bovine bone mineral; CL, cross‐linked; DBBM, deproteinized bovine bone mineral; NCL, non‐cross‐linked.

Under general anesthesia, the maxillary surgical site was shaved and disinfected using betadine solution. Local anesthesia with 0.2% lidocaine hydrochloride‖ and 1:100,000 epinephrine (to ensure hemostasis) was administered. A 40‐mm full‐thickness incision was made at the maxillary bone site, and the subcutaneous tissue and periosteum were carefully dissected bilaterally to expose the maxillary bone. Approximately 7‐mm windows were created in both maxillary sinuses and deliberately perforated (length 5 mm) with a blade. The maxillary SM was meticulously elevated. Four different types of bone graft materials (60 mg), corresponding to the experimental groups, were implanted into the elevated maxillary sinuses. In the DBBM with fibrin glue group, the perforated SM was repaired using fibrin glue before applying the bone substitute. Hemostasis was achieved, and the periosteum and skin were closed in layers using 4‐0 vicryl sutures¶. Postoperative infection and pain were managed by postoperatively administering enrofloxacin # (0.2 mL/kg) and meloxicam** (0.04 mL/kg) intramuscularly. The sutures were removed on postoperative Day 7. Twelve animals were euthanized at 4 and 12 weeks postoperatively (Figure 1).

### 2.5 Microcomputed tomographic analysis

Block sections of the maxillary sinus sites and adjacent tissues were fixed in 10% formalin for 10 days. The fixed specimens were scanned with microcomputed tomography (micro‐CT)†† at 110 kV and 50 µA. The acquired data were processed and reconstructed into 3D images using the NRecon and Dataviewer software‡‡. Cross‐sectional images were used to assess vertical bone gain along the X–Y and Z–Y axes. Bone was defined within a specific gray‐value range based on the histogram analysis. The new bone volume (NBV) within the region of interest (ROI) was determined as a percentage. Several parameters, such as bone volume/total volume (BV/TV), trabecular number (Tb.N), trabecular thickness (Tb.Th), and trabecular separation (Tb.Sp), were evaluated using the CTAn software§§. Additional assessments included total augmentation volume (TAV), NBV, residual bone graft material volume (RMV), and nonmineralized tissue volume (NMV).

### 2.6 Histological analysis

Hematoxylin and eosin (H&E) staining was used to assess general tissue morphology and new bone formation, whereas Masson's trichrome (MT) staining was used to evaluate the presence of residual graft substitutes and connective tissue formation. Micrographs were captured at ×12.5 magnification and subsequently combined to visualize the entire sinus. Histomorphometric analysis was performed using image analysis software‖‖. ROI with dimensions of 0.8 × 0.8 mm were defined to evaluate the distribution of regenerated bone. Three ROI were designated: near the bony window for sinus elevation (ROI_W), at the center of the augmented area (ROI_C), and close to the SM (ROI_M). The relative percentages and values of new bone area (NBA) and residual graft material area (RMA) were calculated for each ROI. The number of vessels in each specimen was recorded. Measurements recorded included the total augmented area (TAA), which represents the area (mm^2^) of the surgical access window; NBA, indicating the percentage (area) of newly formed bone within the TAA; RMA, representing the percentage (area; mm^2^ and %) of bone graft remaining within the TAA; and nonmineralized tissue area (NMA; mm^2^ and %), reflecting the percentage (area) of nonmineralized tissue within the TAA (mm^2^). The resulting H&E and MT staining images were quantified using ImageJ.

### 2.7 Statistical analysis

Data were statistically analyzed using dedicated software¶¶ and presented as mean ± standard deviation (SD) and median values. The experimental results were considered statistically significant at *p *< 0.05.

## 3 RESULTS

### 3.1 In vitro collagen degradation of collagenated bone

**FIGURE 2:**
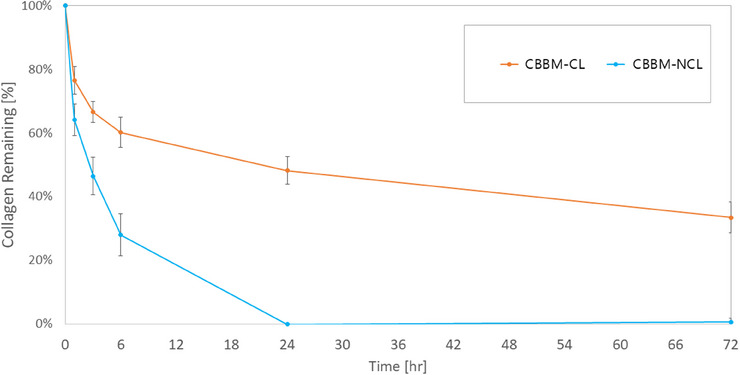
Collagen degradation using collagenase over time. Ten w% collagen content contained in CBBM was converted to 100% in this figure. CBBM, collagenated bovine bone mineral; CL, cross‐linked; NCL, non‐cross‐linked.

The resistance of collagen to collagenase degradation was compared between collagenated bones with the same collagen content. CBBM‐NCL rapidly degraded to approximately 70% of its initial collagen mass within 6 h and was almost completely degraded by 24 h. CBBM‐CL was partially resistant to degradation and retained approximately 33% collagen content, even after 72‐h dissolution. Resistance to collagenase degradation was strongly influenced by cross‐linking, with an initial rapid degradation of collagen in the NCL group and gradual degradation in the CL group (Figure 2 and Table S1–S2 in the online *Journal of Periodontology*).

### 3.2 In vivo experiment

#### 3.2.1 General findings

No adverse events or complications were observed in any of the four groups during the surgical procedure or the healing period.

#### 3.2.2 Volumetric analysis

**FIGURE 3:**
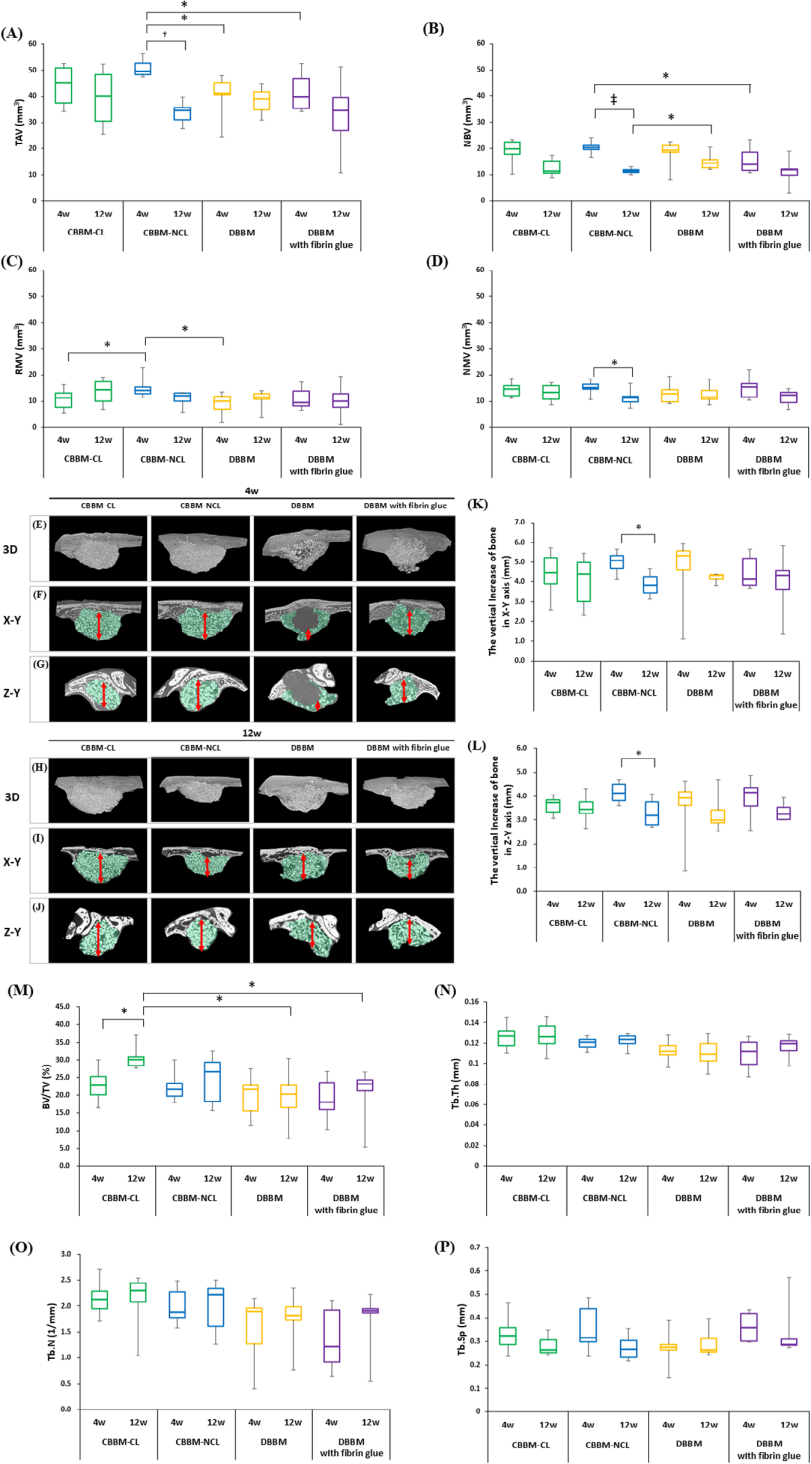
Comparison of TAV at 4 and 12 weeks (A–D). X–Y and Z–Y axes views of augmented area are presented separately. Red arrows show measured distance. Red arrows show the measured vertical height of bone in the X–Y and Z–Y axes, respectively. Statistical analysis of quantitative parameters for micro‐CT and 3D imaging of augmented sinus area (E–L). BV/TV, % (M); Tb.Th, mm (N); Tb.N, mm (O); and Tb.Sp, mm, within augmented sinus (P). Bars with asterisks indicate statistically significant differences. BV/TV, bone volume per unit of total augmented volume; CBBM, collagenated bovine bone mineral; CL, cross‐linked; DBBM, deproteinized bovine bone mineral; micro‐CT, microcomputed tomography; NBV, new bone volume; NCL, non‐cross‐linked; NMV, nonmineralized tissue volume; RMV, residual bone graft material volume; TAV, total augmentation volume; Tb.N, trabecular number; Tb.Sp, trabecular separation; Tb.Th, trabecular thickness. ^*^ *p *< 0.05; ^†^ *p *< 0.01; ^‡^ *p *< 0.001.

Four weeks post TAV, significant differences were observed between CBBM‐NCL and DBBM/DBBM with fibrin glue; in the NBV of CBBM‐NCL compared to that of DBBM with fibrin glue at 4 weeks; and the RMV in the CBBM‐CL versus CBBM‐NCL and CBBM‐NCL versus DBBM groups. At 12 weeks, there was a significant difference in the NBV score between the CBBM‐NCL and DBBM. The TAV, NBV, and NMV of the CBBM‐NCL group at 12 weeks were significantly lower than those at 4 weeks (Figure 3A–D and Table S3 in the online *Journal of Periodontology*). No significant differences were observed in the parameters of all groups at 12 weeks, except for the NBV of CBBM‐NCL compared to DBBM.

#### 3.2.3 Vertical bone increase

Figure 3(E–L) depicts the statistical results and augmented areas in micro‐CT view. Vertical length was defined as the distance between the sinus floor and the maximal augmented point of the SM. For more accurate measurements, the vertical height values in the X–Y and Z–Y axes of the CT image were obtained (see Table S4 in the online *Journal of Periodontology*).

At 4 weeks, vertical bone increases were as follows: CBBM‐CL—4.4  ±  1.1 mm (X–Y) and 3.6 ±  0.4 mm (Z–Y); CBBM‐NCL—5.0  ±  0.6 mm (X–Y) and 4.1 ±  0.4 mm (Z–Y); DBBM—4.6  ±  1.8 mm (X–Y) and 3.5 ±  1.4 mm (Z–Y); DBBM with fibrin glue—4.5  ±  0.9 mm (X–Y) and 3.9 ±  0.8 mm (Z–Y). Both X–Y and Z–Y showed no significant differences across all groups. However, in Z–Y, the comparison between CBBM‐CL and CBBM‐NCL trended toward significance (*p* = 0.070). The volume, size, and shape were similar in the CBBM‐CL and CBBM‐NCL groups; the DBBM and DBBM with the fibrin glue groups were more irregularly shaped than in CBBM‐CL and CBBM‐NCL, and sometimes the bone deviated from the augmented area.

At 12 weeks, dome‐shaped bone formation was observed in all the groups. Irregular shapes were observed in some DBBM and in DBBM with fibrin glue. The values of measurement were as follows: CBBM‐CL—4.1  ±  1.3 mm (X–Y) and 3.5 ±  0.6 mm (Z–Y); CBBM‐NCL—3.9  ±  0.6 mm (X–Y) and 3.3 ±  0.6 mm (Z–Y); DBBM—4.2  ±  0.2 mm (X–Y) and 3.3 ±  0.8 mm (Z–Y); DBBM with fibrin glue—4.0  ±  1.5 mm (X–Y), 3.3 ±  0.4 mm (Z–Y). Neither X–Y nor Z–Y showed any significant differences across all groups. In the CBBM‐NCL group, a significant decrease in X–Y and Z–Y (vertical increase) was observed between Weeks 4 and 12.

#### 3.2.4 Quantitative measurement for bone regeneration

The quantitative results are shown in Figure 3(M–P) and Table S5 in the online *Journal of Periodontology*. At 4 weeks, the BV/TV (%) values were CBBM‐CL—22.9  ±  4.8 mm, CBBM‐NCL—22.4 ±  4.3 mm, DBBM—19.8  ±  6.0 mm, and DBBM with fibrin glue—18.9  ±  6.2 mm. At 12 weeks, the BV/TV (%) values were CBBM‐CL—30.6  ±  3.4 mm, CBBM‐NCL—24.5 ±  7.2 mm, DBBM—19.6  ±  7.6 mm, and DBBM with fibrin glue—20.6  ±  7.7 mm. At 12 weeks, the CBBM‐CL group significantly differed from the DBBM and DBBM with fibrin glue groups (*p *= 0.015 and *p *= 0.018, respectively). The Tb.Th (mm) values of CBBM‐CL versus DBBM and CBBM‐CL vs. DBBM with fibrin glue at 4 weeks, as well as CBBM‐NCL vs. DBBM at 12 weeks, approached statistical significance (*p* = 0.061, *p* = 0.060, and *p* = 0.061, respectively). The difference between Weeks 4 and 12 weeks of CBBM‐CL was statistically significant (*p *= 0.031). The Tb.Sp (mm) value of CBBM‐NCL approached statistical significance between 4 and 12 weeks (*p* = 0.073).

#### 3.2.5 Histomorphometric analysis

**FIGURE 4:**
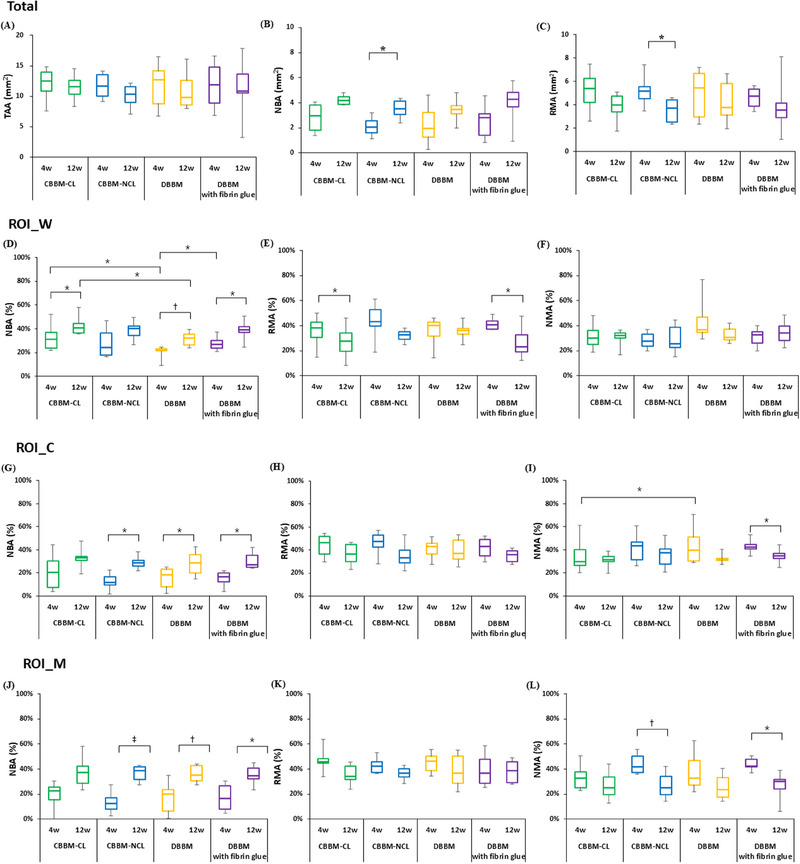
(A–C) Histomorphometric analyses (mean ± SD) of the total area at 4 and 12 weeks; (A) TAA, (B) NBA, (C) RMA. Changes in (D,G,J) NBA, (E,H,K) RMA, and (F,I,L) NMA in ‘ROI_W’, ‘ROI_C’ and ‘ROI_M’ at 4 and 12 weeks. Bars with asterisks indicate significant differences between groups. CBBM, collagenated bovine bone mineral; CL, cross‐linked; DBBM, deproteinized bovine bone mineral; NBA, new bone area; NCL, non‐cross‐linked; NMA, nonmineralized tissue area; RMA, residual graft material area; ROI, region of interest; ROI_C, center region of augmented sinus area; ROI_M, region close to Schneiderian membrane; ROI_W, region close to surgical bony window; SD, standard deviation; TAA, total augmented area. ^*^ *p* < 0.05; ^†^ *p* < 0.01; ^‡^ *p* < 0.001.

There was no significant change in the TAA, NBA, and RMA at 4 and 12 weeks in any of the groups and no significant intergroup relationships at 4 or 12 weeks (Figure 4A–C and Table S6A in the online *Journal of Periodontology*).

The increase in NBA and decrease in RMA were significantly greater in the CBBM‐NCL group than in the other three groups (*p *= 0.010 and *p *= 0.014, respectively).

At 4 weeks, the NBA (%) of ROI_W in all four groups showed higher bone formation than that of ROI_C and M: CBBM‐CL > CBBM‐NCL, DBBM with fibrin glue, and DBBM alone. “CBBM‐CL versus DBBM” and “DBBM versus DBBM with fibrin glue” were significantly different (*p *= 0.041 and *p *= 0.036, respectively), whereas “CBBM‐CL versus DBBM” was the same as at 12 weeks (*p *= 0.020).

ROI_W: The changes in values from 4 to 12 weeks showed significant differences in NBA (CBBM‐CL [*p *= 0.050], DBBM [*p *= 0.005], and DBBM with fibrin glue [*p *= 0.034]) and RMA (CBBM‐CL [*p *= 0.041] and DBBM with fibrin glue [*p *= 0.020]) (Figure 4D–F and Table 6SB–C in the online *Journal of Periodontology*).

ROI_C: “CBBM‐CL versus DBBM” showed a significant difference in NMA at 4 weeks (*p *= 0.003). Significant differences in change between 4 and 12 weeks were observed in NBA, CBBM‐NCL (*p *= 0.016), DBBM (*p *= 0.019), DBBM with fibrin glue (*p *= 0.024), and DBBM with fibrin glue (*p *= 0.011) (Figure 4G–I and Table S6B–C in the online *Journal of Periodontology*).

ROI_M: There was no significant intergroup difference. However, significant differences in NBA and NMA were observed between Weeks 4 and 12 (NBA—CBBM‐NCL [*p *= 0.000], DBBM [*p *= 0.004], and DBBM with fibrin glue [*p *= 0.026]; NMA—CBBM‐NCL [*p *= 0.005] and DBBM with fibrin glue [*p *= 0.017]) (Figure 4J–L and Table 6SB–C in the online *Journal of Periodontology*).

#### 3.2.6 Histologic analysis

**FIGURE 5:**
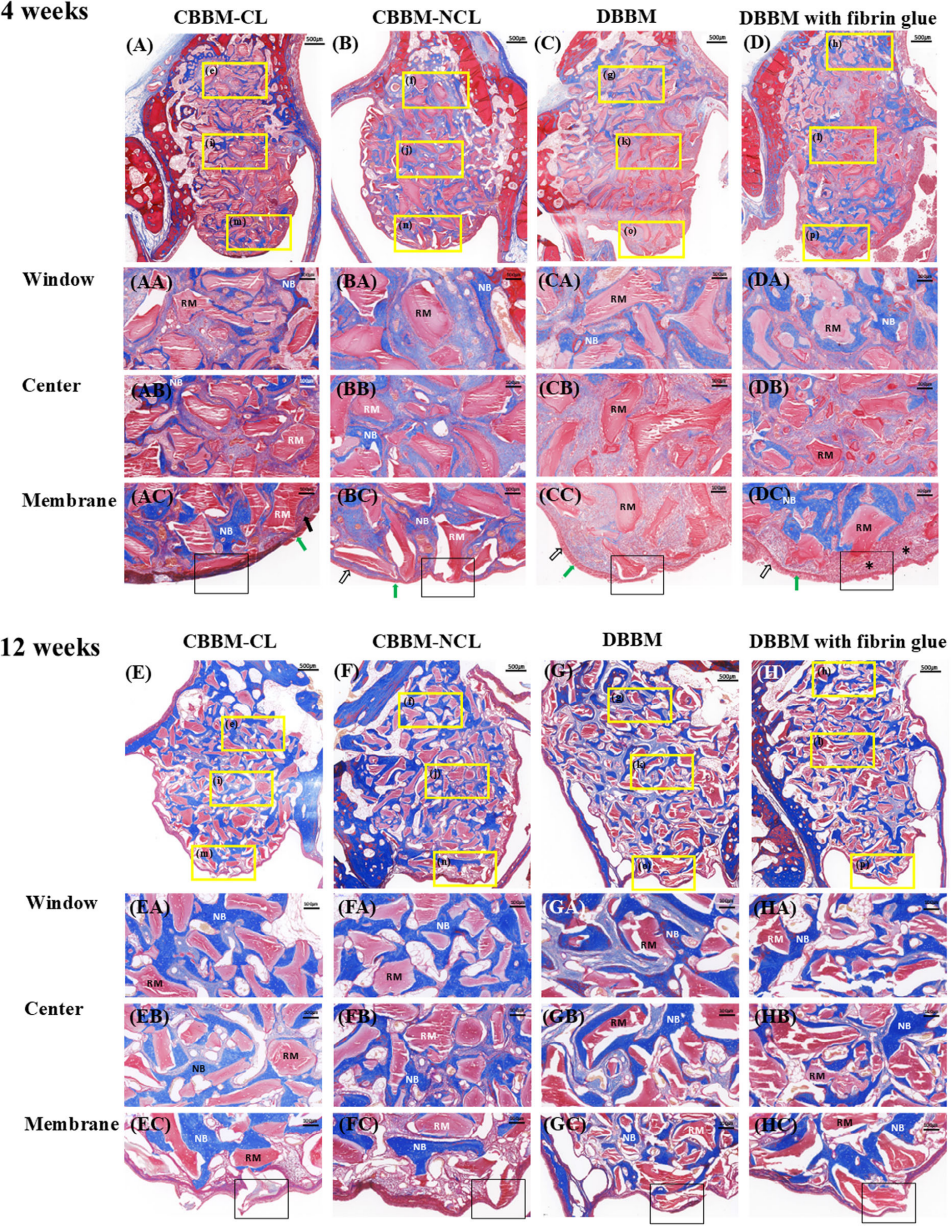
Photomicrograph of MT staining showing sinus augmentation region at 4 and 12 weeks (A–H). Three different areas were chosen in each group for comparison: window (close to bony window), center (in center of elevated space), and membrane (adjacent to SM). Black arrow, serous gland; white arrow, ciliated respiratory epithelium; green arrow, goblet cells; asterisk, connective tissue; square, perforated area. CBBM, collagenated bovine bone mineral; CL, cross‐linked; DBBM, deproteinized bovine bone mineral; MT, Masson's trichrome; NB, newly formed bone; NCL, non‐cross‐linked; RM, residual material; SM, Schneiderian membrane.

Four weeks (Figure 5A–D): In all groups, ciliated epithelium, serous glands, and connective tissue were observed in the nonperforated SM in all groups. However, serous glands were not observed in the perforated SM area, and the bone graft material and ciliated epithelium were in direct contact. CBBM‐CL showed greater ossification than the other three groups. In the DBBM with fibrin glue group, overgrowth of connective tissue and serous glands was observed in the repaired perforation area treated with fibrin glue. Additionally, many blood vessels and bone graft materials were observed within the connective tissue. Ossification near the perforation site was more pronounced in the DBBM with fibrin glue group compared to the DBBM alone group. Lower bone formation was observed in the DBBM group than in the other three groups.

Twelve weeks (Figure 5E–H): Overall, ossification progressed in all groups. The perforated area showed a similar appearance to the fourth week (direct contact between the epithelium and graft material without the serous gland).

#### 3.2.7 Angiogenesis

**FIGURE 6:**
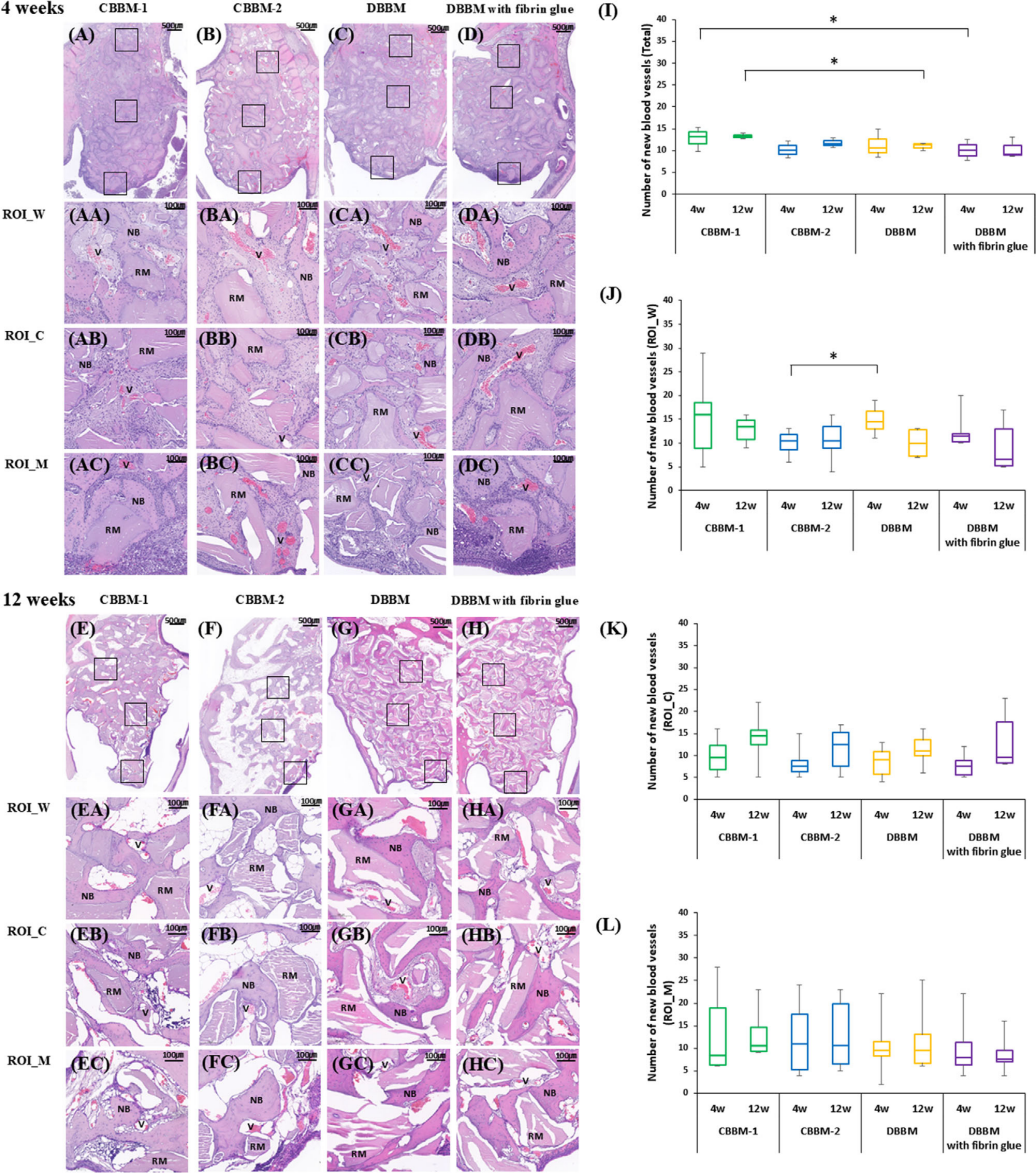
Photomicrographs of H&E staining show sinus augmentation regions at 4 and 12 weeks (A–H) and statistical results (I–L). Neovascularization of different areas. Three different areas were chosen in each group for comparison: ROI_W (close to bony window), ROI_C (in center of elevated area), and ROI_M (adjacent to SM). Numbers of new blood vessels in (I) total area, (J) ROI_W, (K) ROI_C and (L) ROI_M at 4 and 12 weeks. Bars with asterisks indicate significant differences. CBBM, collagenated bovine bone mineral; CL, cross‐linked; DBBM, deproteinized bovine bone mineral; H&E, hematoxylin and eosin; NB, newly formed bone; NCL, non‐cross‐linked; ROI_C, center region of augmented sinus area; ROI_M, region close to SM; ROI_W, region close to surgical bony window; RM, residual material; SM, Schneiderian membrane; V, vessel. ^*^ *p* < 0.05.

The CBBM‐CL group showed a higher total number of new blood vessels than the other groups at 4 and 12 weeks. Significant differences were found between CBBM‐CL and DBBM with fibrin glue (*p *= 0.011) at 4 weeks and between CBBM‐CL and DBBM (*p *= 0.041) at 12 weeks. At 4 weeks in ROI_W, DBBM showed a significantly higher number of vessels than CBBM‐NCL (*p *= 0.014) (Figure 6A–L and Table S7 in the online *Journal of Periodontology*).

## 4 DISCUSSION

This study assessed collagen degradation in CBBM‐CL and CBBM‐NCL in vitro, as well as the outcomes of an in vivo experiment comparing four types of bone substitutes in rabbits with perforated SM. The findings supported the null hypothesis. Volumetric, histomorphometric, and histological analyses were performed to evaluate the augmented area of the maxillary sinus. At 4 weeks, CBBM‐NCL demonstrated significant differences in TAV compared to DBBM and DBBM with fibrin glue, as well as in NBV compared to DBBM with fibrin glue. At 12 weeks, significant differences were observed between CBBM‐NCL and DBBM in NBV. No significant differences in vertical bone increase were observed among groups at 4 and 12 weeks. DBBM and DBBM with fibrin glue exhibited more irregular shapes compared to CBBM‐CL and CBBM‐NCL. In Figure 3(F–G), voids within the maxillary sinus were observed in some cases in DBBM. These voids may result from SM perforation, leading to air infiltration or fluid accumulation.^24^, ^25^ In the BV/TV (%) of 12 weeks, the CBBM‐CL group exhibited statistically significant differences compared to the DBBM and DBBM with fibrin glue groups. In all groups, the value (%) of ROI_W was higher than those of ROI_C and ROI_M. This suggests that new bone formation is more active on the window side due to improved blood supply. In ROI_C, NBA significantly increased in CBBM‐NCL, DBBM, and DBBM treated with fibrin glue. This may be because the ossification progressed from the margins (ROI_W/ROI_M) to the central area. The NBA in ROI_M significantly increased in the CBBM‐NCL, DBBM, and DBBM with fibrin glue groups. This suggests that areas near the SM exhibit higher bone healing potential. This finding is consistent with those of the previous studies.^15^

The purpose of maxillary sinus augmentation is to secure a sufficient maxillary sinus volume for implant placement.^26^ The increased augmented area becomes vulnerable to air pressure in the maxillary sinus. Maintaining volumetric stability of grafted bone substitutes at the augmented site is critical.^27^ Therefore, graft materials with appropriate absorption rates can maintain graft height and are advantageous for sinus augmentation. In a recent meta‐analysis, the incidence of perforation during maxillary sinus augmentation was 29.42%.^28^ The treatment approach varied depending on the size of the perforation. Testori et al. reported that perforations smaller than 5 mm were left to heal naturally, while those measuring 5–10 mm were repaired using biological agents such as collagen membranes or fibrin glue. They also reported that surgery can be postponed for perforations of 10 mm or more that are difficult to repair.^29^ In this study, the perforation length was intentionally 5 mm, unlike in a previous study (3 mm).^15^ This is because a perforation as small as 3 mm may not yield accurate results due to spontaneous healing. When perforation occurs, the collagen membrane is most commonly used for repair. Collagen membranes effectively seal the perforated area, preventing the leakage of graft materials and reducing the risk of sinusitis.^30^ However, collagen membranes have some limitations in clinical use such as (1) difficulty in manipulation, (2) uncertain surgical site visibility, and (3) additional cost of the membrane.^31^ A previous study had attempted the application of CBBM to perforated SM.^15^

CBBM, a soft block bone composed of Type 1 collagen within DBBM, biodegrades within 1–2 weeks of application. Unlike DBBM, CBBM has the advantage of maintaining the shape of the augmented area for a certain period, thereby reducing the risk of bone particle leakage. In this study, the CBBM‐CL and NCL groups had a dome shape of augmented sites, but irregular shapes were observed in the DBBM. The collagen component in CBBM appears to help maintain the shape of the augmented area. A previous study reported that CBBM was helpful in maintaining the volume when used on perforated sinus membranes. However, CBBM is associated with delayed bone healing and volume reduction during collagen biodegradation.^15^ In the histomorphometric results of this study, a marginally significant and significant increase in NBA over time was observed in CBBM‐CL and NCL (*p *= 0.069 and *p *= 0.010, respectively; see Table S6A in the online *Journal of Periodontology*). These findings suggest that (1) collagen in CBBM may impede early bone formation and (2) new bone formation occurs in spaces left by absorbed CBBM collagen.

The results of the in vitro experiments confirmed that the absorption rate was slower in the CL bone (CBBM‐CL) than in the NCL bone (CBBM‐NCL). A previous study has shown that the rapid resorption of collagen followed by decreased mechanical strength is a clinical disadvantage.^16^ In addition, this suggests that the initial rapid degradation of collagen is an excessive immune response that is detrimental to tissue regeneration and reduces stability. The alternative to overcome this shortage is to cross‐link collagen fibers to extend the resorption rate and degradation time.^32^ In the present study, the TAV of CBBM‐NCL was higher than that of CBBM‐CL at Week 4 but lower than that at Week 12. Because of this vertical increase, CBBM‐NCL showed a significant decrease during the healing period. This can be attributed to the initial superior volumetric expansion of CBBM‐NCL compared to CBBM‐CL, followed by a rapid decrease due to collagen degradation over time. In addition, CBBM‐NCL showed a significantly higher level of increased NBA and decreased RMA than CBBM‐CL between 4 and 12 weeks in the histomorphometric result. This suggests that the degradation of collagen provides space for new bone formation, with the newly formed bone occupying the interstitial spaces of the bone graft material. Therefore, it can be concluded that CBBM‐CL may demonstrate superior volumetric stability compared to CBBM‐NCL. Therefore, CBBM‐CL can be seen to have strengths in volumetric stability compared to CBBM‐NCL. At 12 weeks, the volumetric bone gain was similar for both CBBM‐CL and CBBM‐NCL, despite the initial differences observed in the healing process between the two materials. This showed the same results as the previous study as follows: An et al. reported that CL bone blocks tended to have a larger area at the transplant site compared to NCL bone blocks in a rabbit calvaria defect model study.^17^ In this study, the NBV decreased, while the RMV increased from 4 to 12 weeks (see Table S3 in the online *Journal of Periodontology*). These findings contrast with the results of the histomorphometric analysis. One possible explanation is that at 4 weeks, new bone and bone graft materials can be distinguished based on grayscale differences. However, due to the limitations of micro‐CT imaging, these distinctions become less clear by 12 weeks as the grayscale differences between newly formed bone and graft material diminish. Future research should aim to overcome these limitations to improve the accuracy of micro‐CT analysis.^33^

Xin et al. reported that the resorbable collagen membrane in a perforated SM has a sealing effect, but it may delay recovery by disturbing undifferentiated basal cell migration from the surrounding normal area and osteogenic ability.^34^ Just as adding horizontal platelet‐rich fibrin (H‐PRF) to DBBM forms a moldable sticky bone block,^35^ mixing DBBM with fibrin glue creates a soft block bone, helping with bone substitute maintenance. A previous study showed that fibrin glue leads to early wound healing by reducing IL‐1ß and IL‐8 release for 1 week after application.^21^ Buser et al. demonstrated that proinflammatory cytokines such as TNFa, IL‐1ß, IL‐6, and IL‐8 were significantly reduced in encapsulated cells with fibrin glue.^36^, ^37^ In this study, DBBM with fibrin glue showed higher values of NBA followed by DBBM at 4 and 12 weeks. There was more ossification in the DBBM with fibrin glue group than in the DBBM group in the histological images at 4 weeks (Figure 5). It appears that the fibrin component in the fibrin glue can act as a growth factor that helps in early bone formation. Fibrin glue causes excessive growth of connective tissue and serous glands, blocking the outflow of bone particles through the perforation. In addition, due to the influence of fibrin glue, many blood vessels and cells were observed, and it is considered that it may provide a more favorable environment for bone formation in the SM area. This was a preclinical trial. Therefore, the anatomical shape of the sinus, surgical method, and healing period in rabbits differ from those in humans. Additionally, it is essential to consider potential complications associated with collagen‐containing bone graft materials.^38^, ^39^ Further research is required to confirm these findings.

This study has several limitations: (1) The findings may not be applicable in cases where the SM remains intact or when perforations exceed 10 mm in size. (2) The study duration was limited to 12 weeks. Long‐term follow‐up studies of 6 months or more are necessary to confirm these findings.

## 5 CONCLUSION

CBBM for perforated SM repair confers bone stability and ossification. CBBM‐CL was noninferior to CBBM‐NCL in volume stability. The findings indicate that CL collagen in CBBM‐CL contributes to enhanced graft stability over time. Fibrin glue appeared to have a positive effect on early ossification in histological evaluations, but this effect was not evident at 12 weeks.

## AUTHOR CONTRIBUTIONS

**Jung‐Tae Lee**: Conceptualization; execution of experiment; data collection and analysis; writing—original draft. **Seok Hyun Lee**: Data collection and analysis. **Baek Sun Choi**: Data collection and analysis; funding resources. **Sungtae Kim**: Conceptualization; writing—review and editing; supervision; project administration. All authors read and approved the final manuscript.

## CONFLICT OF INTEREST STATEMENT

Seok Hyun Lee and Baek Sun Choi are employed by Osstem Implant Co. Ltd. which supplied materials for this study. All other authors report no conflicts of interest related to this study.

## Supporting information



Supporting Information

Supporting Information

Supporting Information

Supporting Information

Supporting Information

Supporting Information

Supporting Information

Supporting Information

Supporting Information

## Data Availability

All datasets generated and/or analyzed during the present study are available from the corresponding author on reasonable request.

## Footnotes

* A‐Oss Collagen; Osstem Implant Co. Ltd., Seoul, Republic of Korea.

† Bio‐Oss Collagen; Geistlich Pharma AG, Wolhusen, Switzerland

‡ A‐Oss; Osstem Implant Co. Ltd., Seoul, Republic of Korea

§ A‐Oss with Tisseel; Baxter Healthcare Co. Ltd., Deerfield, Illinois, USA

‖ Ketalat; Yuhan, Seoul, Republic of Korea

¶ Ethicon, Somerville, New Jersey, USA

# Baytril; Elanco, Greenfield, Indianapolis, USA

** Metacam; Boehringer, Ingelheim, Germany

†† InspeXio SMX‐225 CT; Shimadzu, Kyoto, Japan

‡‡ Bruker MicroCT; Bruker, Kontich, Belgium

§§ Bruker, Kontich, Belgium

‖‖ 3DHISTECH Ltd., Budapest, Hungary

¶¶ MedCalc Software Ltd., Ostend, Belgium
