# Supplementary figures and images for: Comparison of four bone substitute types in sinus augmentation with perforated Schneiderian membrane: An experimental study
Source: J Periodontol. 2025 Apr 2;96(10):1154–67. doi: 10.1002/JPER.24-0663 (PMC12572692; doi:10.1002/JPER.24-0663)

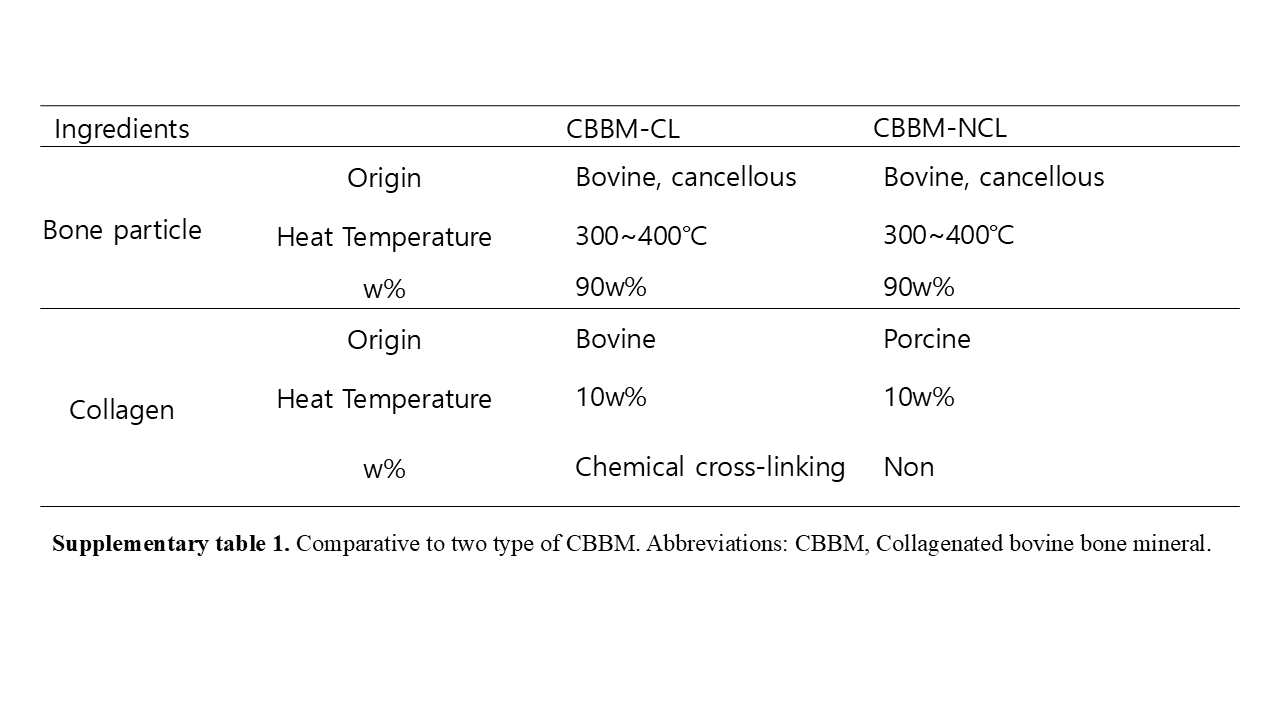

Supplement: Supplementary file 1 — Supporting Information [file JPER-96-1154-s007.tif]

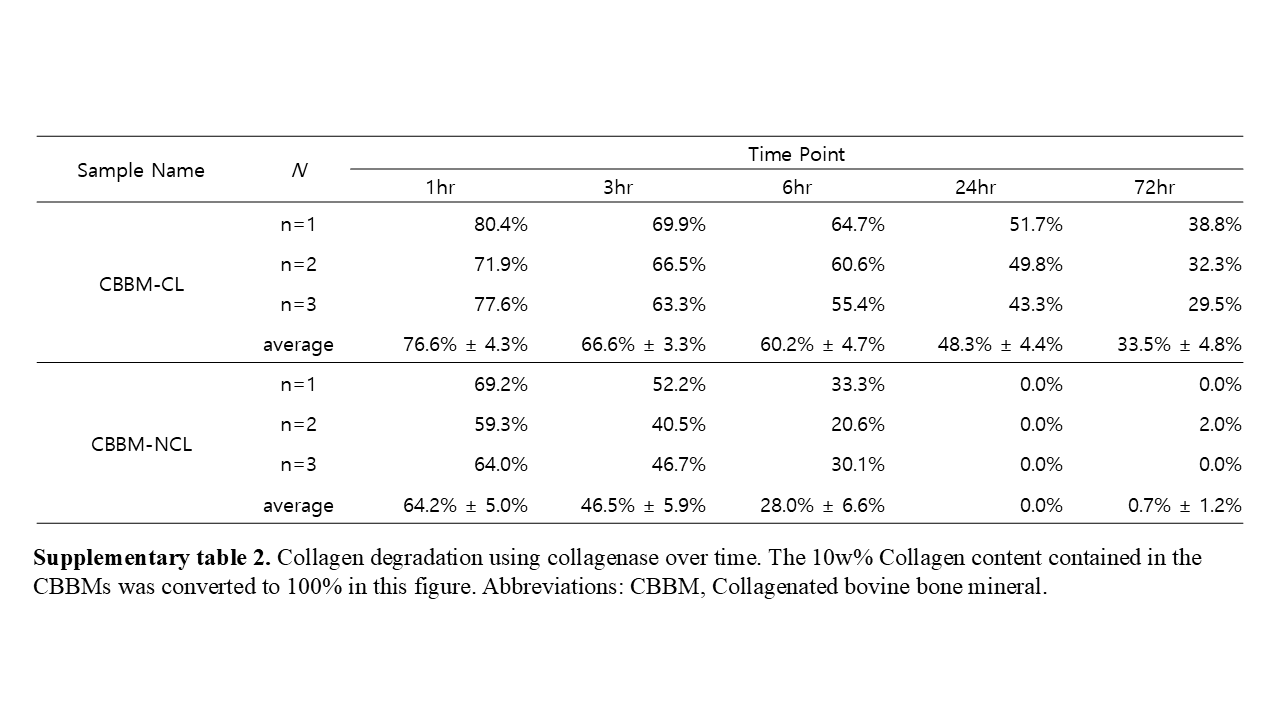

Supplement: Supplementary file 2 — Supporting Information [file JPER-96-1154-s002.tif]

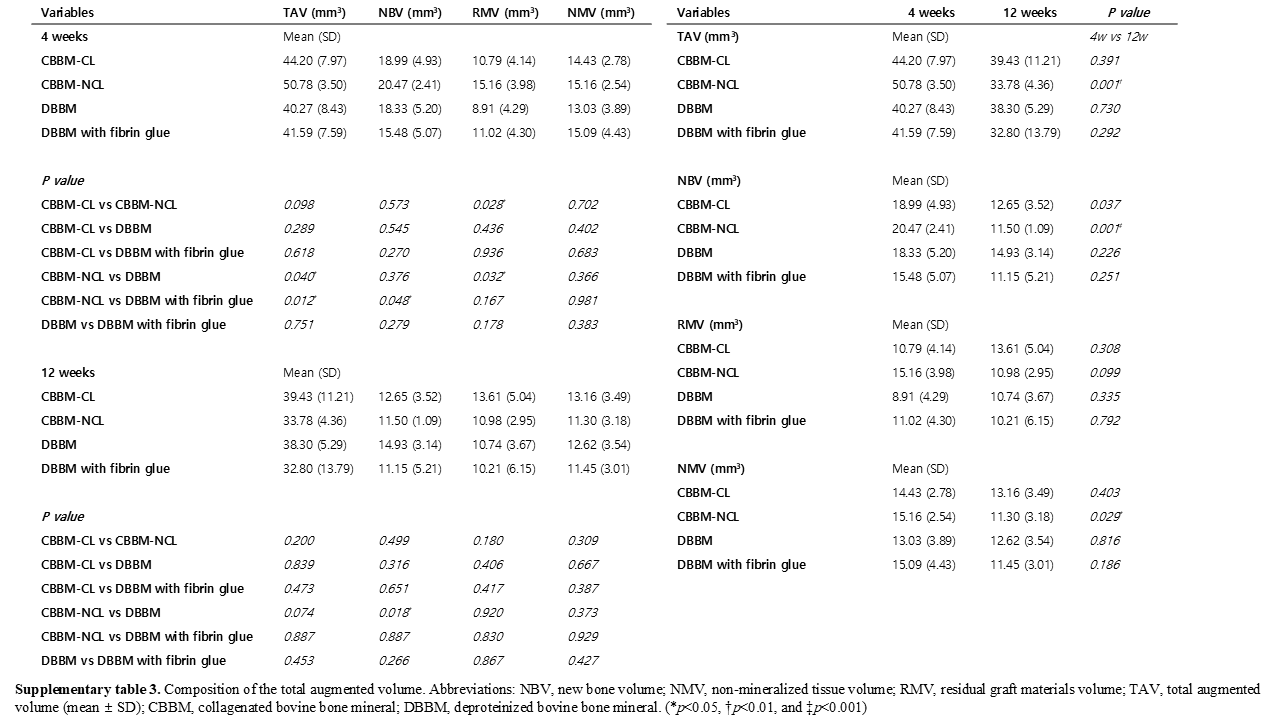

Supplement: Supplementary file 3 — Supporting Information [file JPER-96-1154-s008.tif]

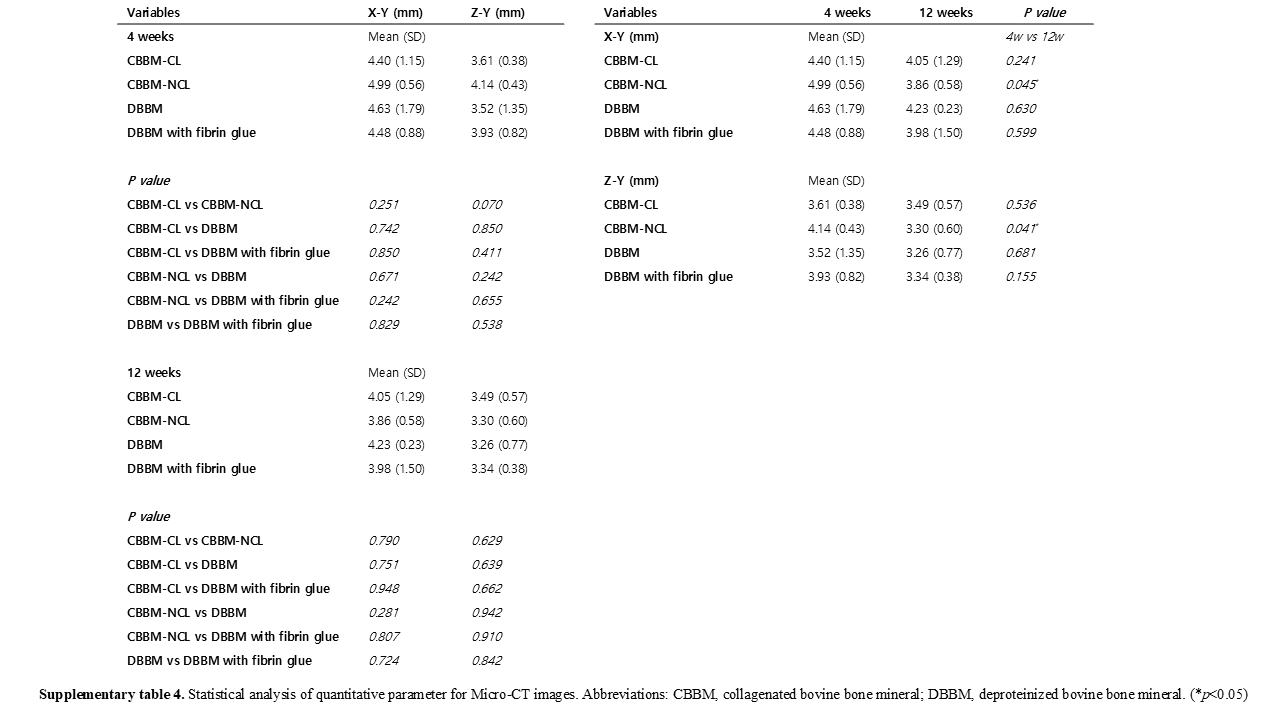

Supplement: Supplementary file 4 — Supporting Information [file JPER-96-1154-s005.tif]

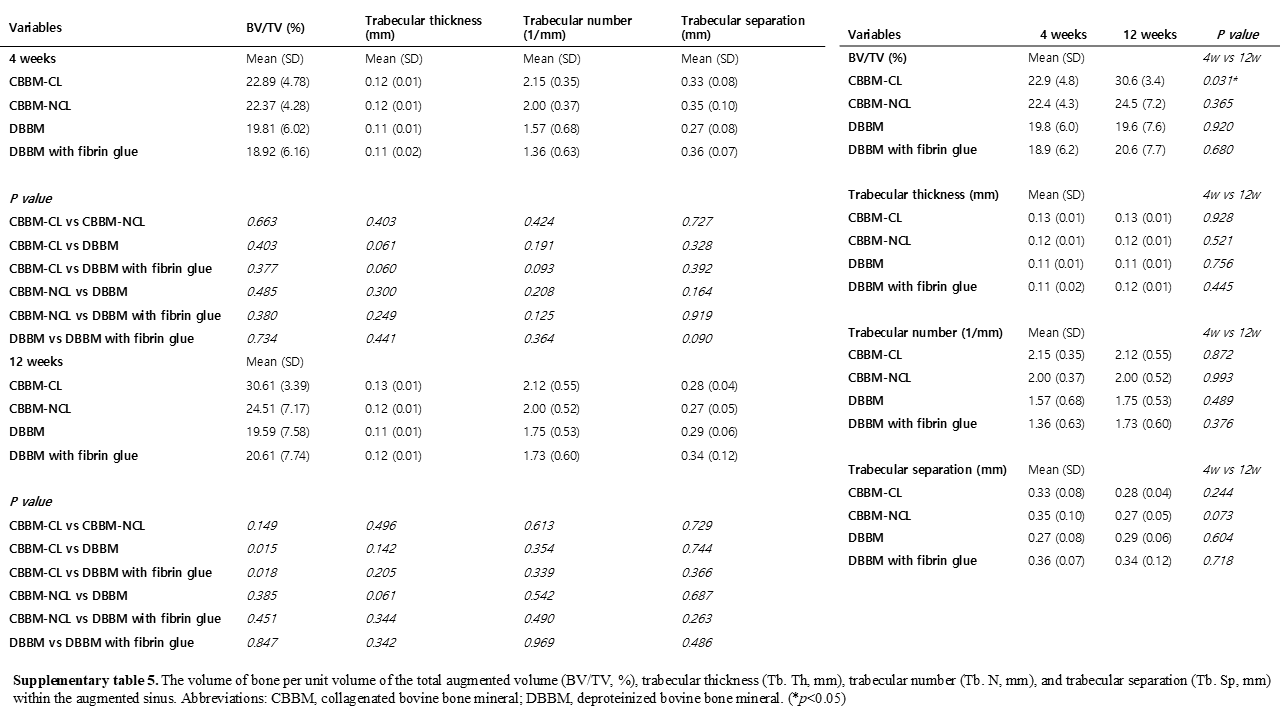

Supplement: Supplementary file 5 — Supporting Information [file JPER-96-1154-s009.tif]

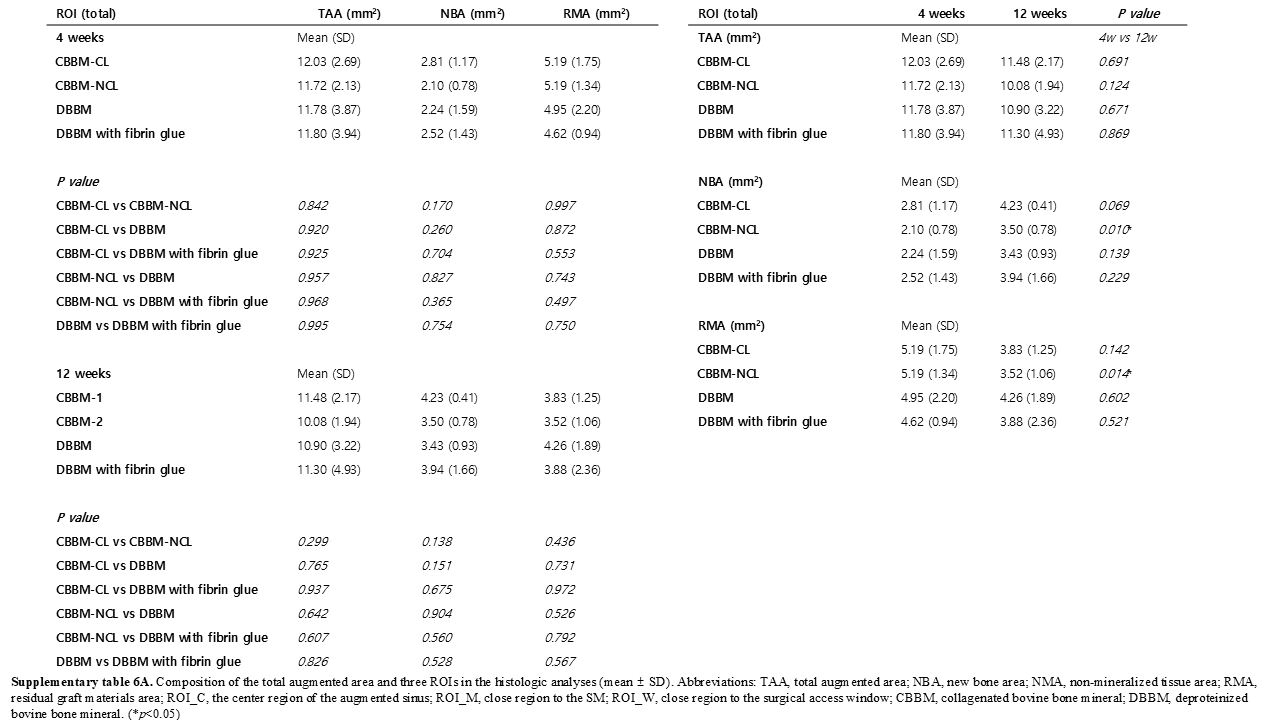

Supplement: Supplementary file 6 — Supporting Information [file JPER-96-1154-s003.tif]

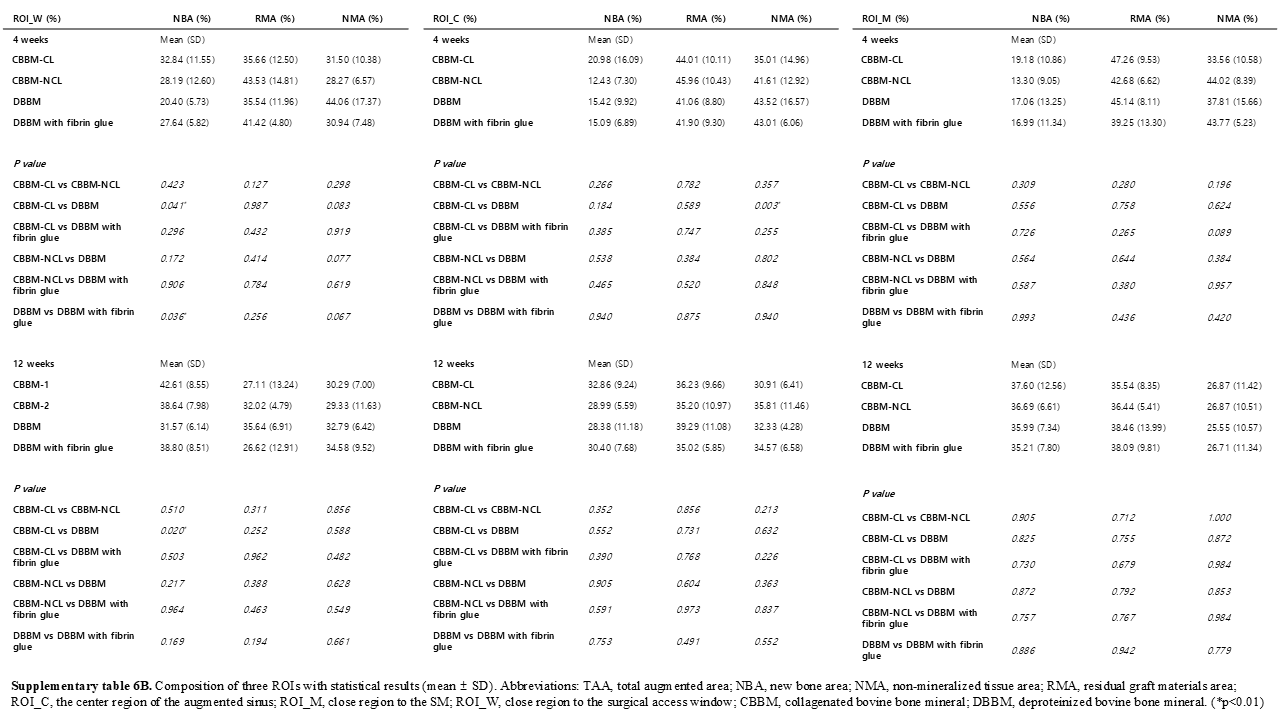

Supplement: Supplementary file 7 — Supporting Information [file JPER-96-1154-s001.tif]

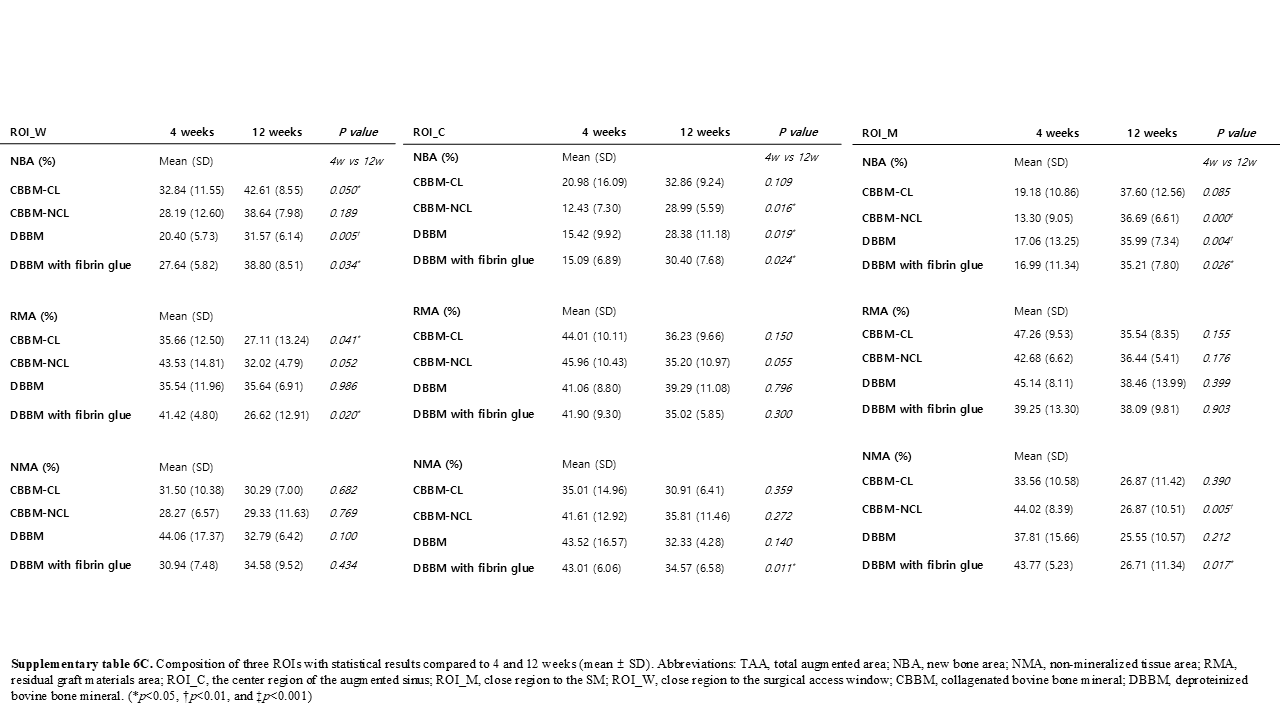

Supplement: Supplementary file 8 — Supporting Information [file JPER-96-1154-s004.tif]

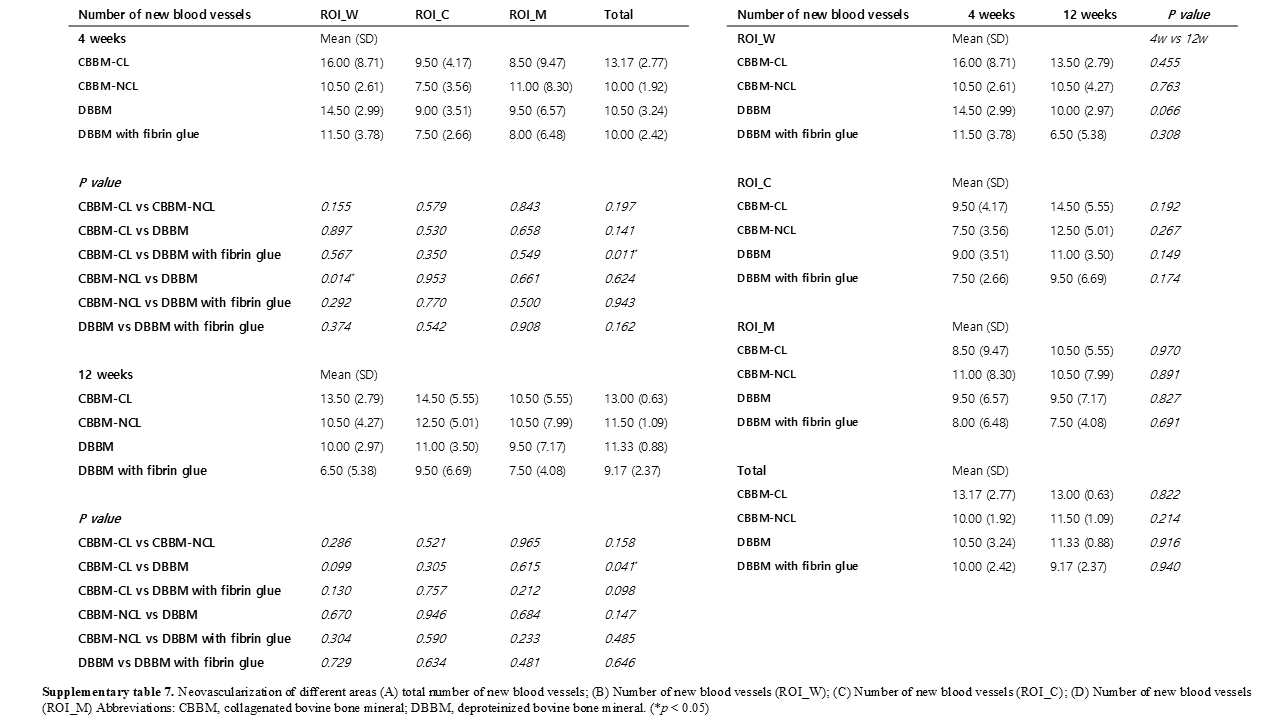

Supplement: Supplementary file 9 — Supporting Information [file JPER-96-1154-s006.tif]
